# Berberine Regulates Treg/Th17 Balance to Treat Ulcerative Colitis Through Modulating the Gut Microbiota in the Colon

**DOI:** 10.3389/fphar.2018.00571

**Published:** 2018-05-31

**Authors:** Huantian Cui, Yuzi Cai, Li Wang, Beitian Jia, Junchen Li, Shuwu Zhao, Xiaoqian Chu, Jin Lin, Xiaoyu Zhang, Yuhong Bian, Pengwei Zhuang

**Affiliations:** ^1^Tianjin University of Traditional Chinese Medicine, Tianjin, China; ^2^Tianjin Second People’s Hospital, Tianjin, China

**Keywords:** ulcerative colitis, berberine, gut microbiota, Treg/Th17 balance, inflammation

## Abstract

Berberine (BBR), an alkaloid isolated from *Rhizoma Coptidis, Cortex Phellode*, and *Berberis*, has been widely used in the treatment of ulcerative colitis (UC). However, the mechanism of BBR on UC is unknown. In this study, we investigated the activities of T regulatory cell (Treg) and T helper 17 cell (Th17) in a dextran sulfate sodium (DSS)-induced UC mouse model after BBR administration. We also investigated the changes of gut microbiota composition using 16S rRNA analysis. We also examined whether BBR could regulate the Treg/Th17 balance by modifying gut microbiota. The mechanism was further confirmed by depleting gut microbiota through a combination of antibiotic treatment and fecal transplantations. Results showed that BBR treatment could improve the Treg/Th17 balance in the DSS-induced UC model. BBR also reduced diversity of the gut microbiota and interfered with the relative abundance of *Desulfovibrio, Eubacterium*, and *Bacteroides.* Moreover, BBR treatment did not influence the Treg/Th17 balance after the depletion of gut microbiota. Our results also revealed that fecal transplantation from BBR-treated mice could relieve UC and regulate the Treg/Th17 balance. In conclusion, our study provides evidence that BBR prevents UC by modifying gut microbiota and regulating the balance of Treg/Th17.

## Introduction

Ulcerative colitis (UC) is a chronic and nonspecific inflammatory disease that has a prolonged disease duration and is difficult to cure. The morbidity of UC has increased over the past few years. Long term effects of UC could lead to cancer (Matsunaga et al., 2017).

Currently, the pathogenesis of UC remains unclear. Hereditary factors, the gut environment, immune response, cell apoptosis, and infection have been implicated in UC. During UC progression, the T helper cell 17 (Th17) population, which contributes to inflammation, is usually increased, while T regulatory cells (Treg), which inhibit Th17 activity, are decreased (Luo et al., 2017). UC patients exhibit gut microbiota dysfunction which results in increased permeability of the intestinal epithelial cell barrier, triggering inflammatory response of the intestine (Hoque et al., 2000). Furthermore, probiotics, which could regulate the immune function and promote intestinal mucosa repair, are decreased in UC patients (Shen et al., 2014). Therefore, modulating gut microbiota to promote intestinal mucosa repair is of clinical importance in UC studies (Cui et al., 2004; Zhao H.M. et al., 2013).

Adrenocortical hormone, 5-amino salicylic acid, and immunosuppressive drugs are commonly used in the current treatment of UC. However, these drugs have serious side-effects including intolerance or allergy responses (Di Paolo et al., 2001). Some medicinal plants have been demonstrated to have protective effects on UC. Microwave-assisted aqueous harpagophytum extract and hydroalcoholic extract of chamomile could relief UC through modulating the inflammatory response in a rat colon inflammatory model (Menghini et al., 2016; Locatelli et al., 2017). Traditional Chinese medicine (TCM) is widely used in the treatment of UC. Accumulated studies have shown that TCM plays an important role in regulating the gut microbiota during UC treatment. Gancaoxiexin decoction has been shown to increase the numbers of *Lactobacillus* and *Bifidobacterium*, and decrease the numbers of *Escherichia coli* in the intestine of UC patients (Zhao Q. et al., 2013). Interleukin (IL*)*-10 is produced by Treg and could suppress inflammatory response in UC (Jonuleit and Schmitt, 2003). Whereas *IL-33* is produced by colonic epithelial cells and could drive a Th2-like cytokine response in UC patients (Seidelin et al., 2010). *Tumor Necrosis Factor (TNF)-α* is a proinflammatory cytokine and its level increased in UC patients (Rijnierse et al., 2006). Yiqishengyu decoction demonstrated a significant effect on UC by improving the microenvironment imbalance and modulating the *IL-10* and *IL-33* levels (Yao et al., 2013). *Portulaca oleracea* polysaccharide has also been shown to increase the amount of *Lactobacillus* and *Bifidobacterium*, increase the levels of *IL-10*, and decrease the *TNF-α* levels in the intestine of UC modelmice (Feng et al., 2015).

Berberine (BBR), an alkaloid isolated from *Rhizoma Coptidis, Cortex Phellode*, and *Berberis*, exhibits many biological functions and has been used in the treatment of diarrhea, gastroenteritis, diabetes, hyperlipidemia, cardiovascular diseases, and UC (Chen et al., 2014). Interestingly, it has been demonstrated that BBR prevents obesity and insulin resistance by modulating the gut microbiota (Zhang et al., 2012). Moreover, studies have demonstrated that probiotics such as *Bifidobacterium fragilis* could improve the mucosal immune system in UC through interaction with intestinal regulatory T cells (Treg) to induce *IL-10* production (Smith et al., 2013; Luo et al., 2017). Based on these lines of evidence, we hypothesized that BBR could modulate the gut microbiota and regulate the Treg/Th17 balance in UC. In this study, we first investigated whether BBR could influence the Treg/Th17 balance in UC model mice. We then investigated the changes of gut microbiota composition using 16S rRNA analysis after BBR treatment. Gut microbiota were depleted using a combination of antibiotics. Fecal transplantations were used to further confirm that BBR regulates the Treg/Th17 balance to prevent UC by modulating the gut microbiota.

## Materials and Methods

### Reagents

The standard of berberine (C_20_H_18_NO_4_, molecular weight: 235.324 kD) was obtained from Solarbio Biotechnology Co., Ltd. (Beijing, China). Dextran sulfate sodium (DSS molecular weight: 36000-50000kD), ciprofloxacin, metronidazole, Phorbol 12-myristate (PMA), ionomycin, and Bravertin A were also obtained from Solarbio Biotechnology Co., Ltd. (Beijing, China). Total DNA extraction kit, total RNA extraction kit, first-stand cDNA reverse transcription kit, polymerase chain reaction kit, and primers were obtained from TianGen Biotechnology Co., Ltd. (Beijing, China). Mouse IL-10 and IL-17 ELISA kits were obtained from Multi Science Biotechnology Co., Ltd. (Hangzhou, China). APC anti-CD4, FITC anti-IL17A, PE anti-CD25, and Alexa Fluor 488 anti-Foxp3 antibodies for flow cytometry were purchased from BD bioscience Co., Ltd. (Franklin Lakes, NJ, United States). BCA, alanine aminotransferase (ALT), aspartate aminotransferase (AST), blood urea nitrogen (BUN), and creatinine (Cr) test kit were purchased from Nanjing Jiancheng Biological Engineering Institute (Nanjing, China).

### Animals

Thirty- to forty-day-old female Balb/c mice (18–22 g), were purchased from Beijing Huafukang Animal Company. All animals were handled using experimental protocols outlined by National Institutes of Health regulations and approved by the Ethics Committee and Use Committee of the Tianjin University of Traditional Chinese Medicine. Throughout the acclimatization and study periods, all animals had access to food and water *ad libitum* and were maintained on a 12 h light/dark cycle (21 ± 2°C with a relative humidity of 45 ± 10%).

### Induction of UC Using DSS

Ulcerative colitis was induced by administrating mice with 4% (w/v) DSS in the drinking water for 7 days. Thereafter, mice were provided with regular water for 3 days (Wang et al., 2016).

### Depletion of the Gut Microbiota

Mice were given a combination of ciprofloxacin (0.2 g/l) and metronidazole (1 g/l) for 3 weeks in their drinking water (Elinav et al., 2011). Fecal samples were collected, and the total DNA of the samples was assessed for gut microbiota depletion. Liver tissues were weighed to measure the liver index. In addition, liver, kidney, small intestine, and colon were stained with hematoxylin and eosin (H&E) and serum levels of AST, ALT, Cr, and BUN were also measured to evaluate the effect of antibiotic treatment on liver and kidney function.

### Animal Grouping

Mice were randomly divided into three groups (*n* = 10 per group): DSS-BBR-, DSS+BBR-, and DSS+BBR+. The DSS+BBR- and DSS+BBR+ groups received DSS diluted in drinking water to induce UC. Mice in the DSS+BBR+ group were treated with BBR (40 mg/kg) whereas mice in the DSS-BBR- group and DSS+BBR- group were treated with 0.2 mL normal saline orally once per day.

For the gut microbiota depletion experiment, mice were randomly divided into three groups (*n* = 10 per group): antibiotics-DSS-BBR-, antibiotics+DSS+BBR-, and antibiotics+DSS+BBR+. Mice in the antibiotics-DSS-BBR- group remained untreated; mice in the antibiotics+DSS+BBR- and antibiotics+DSS+BBR+ groups were treated with a combination of antibiotics to deplete the gut microbiota followed by administration of DSS diluted in drinking water to induce UC. Meanwhile, mice in the antibiotics+DSS+BBR+ group were treated with BBR (40 mg/kg) orally once per day during DSS treatment whereas mice in the antibiotics-DSS-BBR- and the antibiotics+DSS+BBR- groups were treated with 0.2 mL normal saline orally during DSS treatment.

**FIGURE 1:**
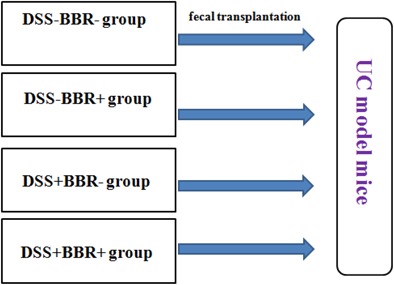
Group of fecal transplantation experiment. Fecal samples of DSS–BBR–, DSS–BBR+, DSS+BBR– and DSS+BBR+ treated mice were transplanted to DSS-induced UC model mice.

The fecal transplantation experiment was performed based on an established protocol (Borody et al., 2013) (**Figure 1**). In brief, donor mice were randomly divided into four groups (*n* = 6 per group) including DSS-BBR-, DSS-BBR+, DSS+BBR-, and DSS+BBR+. The DSS-BBR- group was fed normally; the DSS-BBR+ group received BBR treatment (40 mg/kg) orally once per day; and the DSS+BBR- and DSS+BBR+ groups received DSS diluted in drinking water to induce UC. The DSS+BBR+ group received BBR treatment (40 mg/kg) orally once per day whereas the DSS+BBR- group received 0.2 mL normal saline treatment. After 10 days, stools from each group were collected daily from donor mice for the subsequent 10 days under a laminar flow hood in sterile conditions. Stools from donor mice of each group were pooled and 100 mg stools were re-suspended in 1 ml of sterile normal saline. The solution was vigorously mixed for 10 s using a bench top vortex followed by centrifugation at 800 *g* for 3 min. The supernatant was collected and used as transplant material as described below. Fresh transplant material was prepared on the same day of transplantation within 10 min before oral gavage to prevent changes in bacterial composition. Recipient mice were randomly divided into four groups (*n* = 7 per group). Each group of recipient mice received DSS treatment to induce UC and 100 μl of fresh transplant material orally per day for 10 days.

### Disease Activity Index (DAI)

The severity of UC in each group was measured using DAI score. DAI is the combined score of body weight loss, stool consistency, and rectal bleeding, as in **Table 1** and described previously (Kihara et al., 2003). The scores of DAI ranged from 0 (healthy) to 4 (severe colitis). The historical score is correlated with the pathologic findings in the DSS-induced model.

**Table 1:** Disease activity index (DAI) (Kihara et al., 2003).

Score	Weight loss	Stool consistency	Rectal bleeding
0	<1%	Normal	Negative
1	5–10%		
2	10–15%	Loose stool	Hemoccult+
3	15–20%		
4	>20%	Diarrhea	Gross bleeding

*Clinical criteria were used to evaluate the grade and extent of intestinal inflammation. Scores were tallied for each category and then divided by three to obtain the DAI.*

### Histology

Mice liver, spleen, kidney, small intestine, and colon were removed and fixed in paraformaldehyde. The colon was then paraffin embedded and subsequently cut into 5 μM sections. Sections were stained with hematoxylin and eosin (H&E), the extent of inflammation was determined through a histological colitis score based on a previous study (Kihara et al., 2003). Scores ranged from 0 to 14 (total score), which represents the sum of scores from 0 to 4 for severity of extent, damage, inflammation, and regeneration (**Table 2**).

**Table 2:** Histological colitis score (Kihara et al., 2003).

Feature score	Score	Description
Inflammation severity	0	None
	1	Mild
	2	Moderate
	3	Severe
Inflammation extent	0	None
	1	Mucosa
	2	Submucosa
	3	Transmural
Crypt damage	0	None
	1	Basal 1/3 damage
	2	Basal 2/3 damage
	3	Crypt lost; surface epithelium present
	4	Crypt and surface epithelium lost
Percent involvement	0	0%
	1	1–25%
	2	26–50%
	3	51–75%
	4	76–100%

*The severity of inflammation was evaluated by histological colitis score. The score ranged from 0 to 14 (total score), which represents the sum of scores from 0 to 4 for severity and extent of inflammation, crypt damage, and percent of the colon involved.*

### Cytokine Quantification by Enzyme-Linked Immunoassay (ELISA)

Colon tissue (0.1 g) was weighed and put into 900 μl normal saline followed by ultrasonic trituration and centrifugation at 3000 rpm for 15 min to obtain colon tissue homogenate. The levels of *IL-10* and *IL-17* in the colon tissue homogenate were measured by ELISA according to the manufacturer’s instructions (Multi Science Biotechnology Co., China). Tissue homogenates were normalized to total protein content as detected by BCA assay (Nanjing Jiancheng Bioengineering Institute). The absorbance value was detected using a microplate reader.

### RNA Isolation and Real-Time Reverse Transcription Quantitative Polymerase Chain Reaction (RT-PCR)

Total RNA was isolated from mice colon mucosa using the RNA extraction kit and first strand cDNA was synthesized from 1 μg of total RNA according to the manufacturer’s instructions. Quantitative RT-PCR (qRT-PCR) was used to detect the expression of *β-actin, IL-10, IL-17, Fox3p*, and *RORγt* as previously described (Citri et al., 2011). All samples were run in triplicate and detected using a BIORAd iQ5 detection system. *β-actin* was used as a loading control. Quantification was undertaken using the 2^-ΔΔC_T_^ method (Livak and Schmittgen, 2001). The sequences of all primers are listed in **Table 3**.

**Table 3:** Primer sequences of target genes for mice.

Genes	Primer sequence (5′-3′)	Amplicon size (bp)
*β-actin*	Forward: GCT GTC CCT GTA TGC CTC T	461
	Reverse: GGT CTT TAC GGA TGT CAA CG	
IL-10	Forward: TAA TAA GCT CCA AGA CCA AG	262
	Reverse: TAG AAT GGG AAC TGA GGT ATC	
IL-17	Forward: GTC AAT GCG GAG GGA AAG	349
	Reverse: CAC GAA GCA GTT TGG GAC	
Foxp3	Forward: CAG GAG AAA GCG GAT ACC AAA TG	366
	Reverse: ATC TGT GAG GAC TAC CGA GCC	
RORγT	Forward: ACC TCC ACT GCC AGC TGT GTG CTG TC	440
	Reverse: TCA TTT CTG CAC TTC TGC ATG TAG ACT GTC CC	

### Flow Cytometry

Spleens were placed in 5 mL lymphocyte separation solution after mice were sacrificed. Single-cell suspensions were prepared by grinding over a nylon membrane (70-μm pore size), and lymphocytes were isolated after centrifuging at 800 *g* for 30 min. The isolated cells were collected and treated with PMA (30 ng/mL), ionomycin (1 μg/mL), and BFA (10 ng/mL) for 4 h. Standard intracellular cytokine staining was used as previously described (Harrington et al., 2005). APC anti-mouse CD4 and FITC anti-mouse IL-17A were used for Th17 cell staining, whereas APC anti-mouse CD4, PE anti-mouse CD25, and Alexa Fluor 488 anti-mouse Foxp3 were selected for Treg staining. Samples and data were collected and analyzed with a BD-related device and software (FACSCalibur and Cell Quest-Pro).

### Isolation of Total DNA From Feces

Fresh stool samples were collected and weighed. Total DNA in stool was isolated using the DNA extraction kit (TIANGEN, China). The DNA in stools was quantified as previously described (Li et al., 2017).

### Fecal 16S rRNA Analysis

DNA was diluted to 1 ng/μL using sterile water. The extracted DNA from each sample was used as template to amplify the V4 region of 16S rRNA genes of distinct regions (16SV4) with specific primers (515F: 5′-GTGCCAGCMGCCGCGGTAA-3′ 806R: 5′-GGACTACHVGGGTWTCTAAT-3′). All PCR reactions were carried out using Phusion^®^High-Fidelity PCR Master Mix (New England Biolabs). PCR products were mixed with same volume of 1X loading buffer (containing SYBR green) and detected with electrophoresis on a 2% agarose gel. Further experiments were conducted using samples with bright bands between 280 and 320 bp. The TruSeq^®^DNA PCR-Free Sample Preparation Kit (Illumina, United States) was used to generate sequencing libraries. The library quality was assessed on the Qubit@ 2.0 Fluorometer (Thermo Scientific) and Agilent Bioanalyzer 2100 system. Finally, the library was sequenced on a Thermo Fisher Scientific Ion S5 XL platform and 600 bp single-end reads were generated.

### Statistical Analysis

All data were analyzed using one way analysis of variance (ANOVA) followed by Newman–Keuls comparison multiple test with mean ± standard deviation (mean ± SD) for the independent experiments. Statistical differences between the experimental groups and control were examined and statistical significance was determined at a *p*-value < 0.05, using SPSS version 20.0. Curve-fitting was carried out using GraphPad Prism5.

## Results

### BBR Improved Treg/Th17 Balance in DSS-Induced UC Model Mice

**FIGURE 2:**
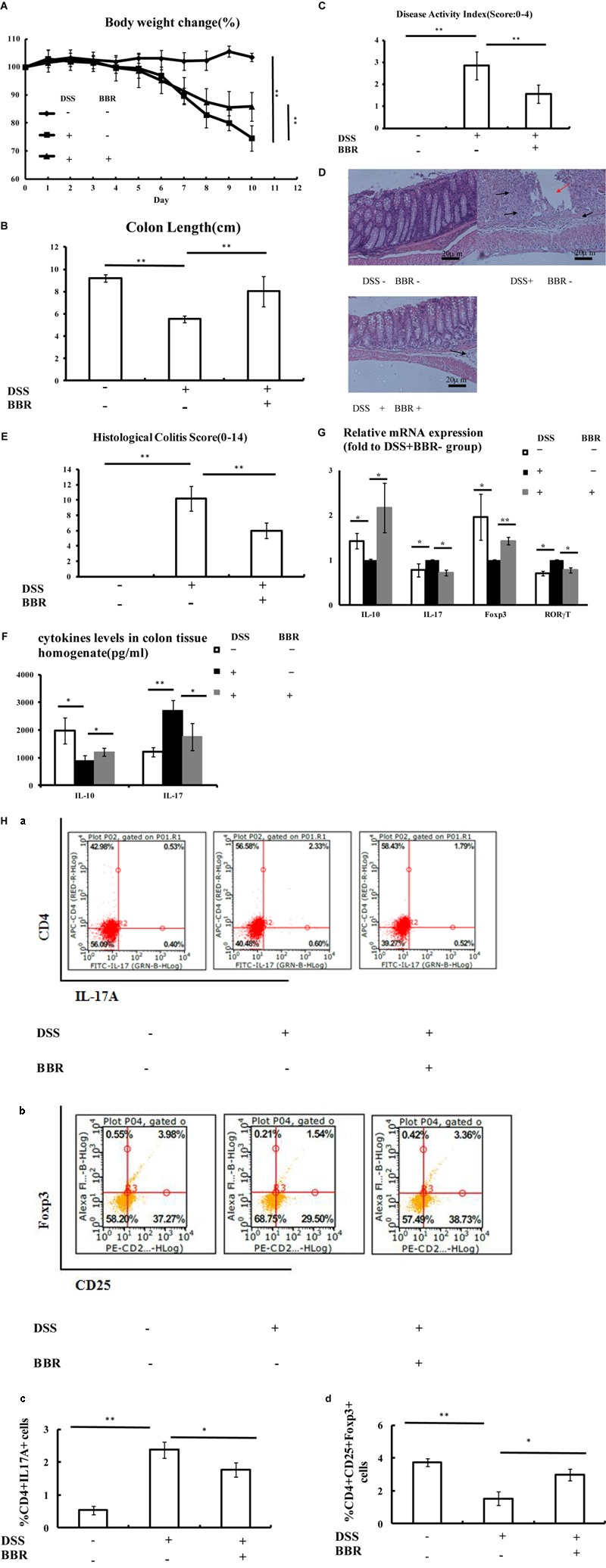
Berberine (BBR) improved Treg/Thl7 balance in DSS-induced UC model mice. **(A,B)** Body weight loss and shorten of colon length were reduced after BBR treatment in DSS-induced UC model mice; **(C)** Mice treated with BBR had a lower DAI score compared with DSS treated mice; **(D,E)** H&E staining (Arrows in red indicate mucosal necrosis, arrows in black indicate inflammatory cell infiltration; 20x) and histological colitis score showed that BBR reduced mucosal necrosis and inflammatory cell infiltration in colon; **(F)** BBR treatment increased *IL-10* and decreased *IL-17* levels of colon tissue homogenate in DSS-induced UC model mice; **(G)** After BBR treatment, the relative mRNA expression of *IL-10* and *Foxp3* in colonic mucosa was upregulated whereas the relative mRNA expression of *IL-17* and *RORγT* in colonic mucosa was downregulated as compared with DSS+BBR– group (Fold to DSS+BBR– group); (**H**) Flow cytometric analysis showed that BBR treatment decreased the numbers of CD4^+^IL17A^+^ (**a**: plot; **c**: graph) cells and increased the numbers of CD4^+^CD25^+^Foxp3^+^ (**b**: plot; **d**: graph) in spleen compared with DSS+BBR– group. DSS–BBR–; DSS+BBR–; DSS+BBR+ (*n* = 10 per group, for **A–G)**. DSS–BBR–; DSS+BBR–; DSS+BBR+ (*n* = 6 per group, **H**). Data are presented as mean ± SD. ^∗^*p* < 0.05, *^∗∗^p <* 0.01.

Ulcerative colitis developed in all mice after DSS administration, evidenced by continuous body weight loss, shortened colon length, and high DAI score (**Figures 2A–C**, respectively) compared with animals without DSS treatment (*P* < 0.01, *P* < 0.05, *P* < 0.01, respectively). Remarkably, BBR treatment reduced body weight loss and decreased loss of colon length caused by DSS administration, shown by the fact that mice treated with BBR had a lower DAI score (*P* < 0.01). H&E staining also showed that BBR relieved mucosal necrosis and infiltration of inflammatory cells triggered by DSS treatment, which resulted in lower histological colitis score compared with the model group (*P* < 0.05, **Figures 2D,E**). Treg and Th17 activities were measured in each group. *IL-10* levels in colonic tissue homogenate were decreased while *IL-17* levels were increased in the DSS + BBR- group compared to the DSS-BBR- group (*P* < 0.05, *P* < 0.01, respectively **Figure 2F**). BBR treatment increased *IL-10* levels in colonic tissue homogenate and decreased colonic *IL-17* levels compared to mice treated with DSS alone (*P* < 0.05, **Figure 2F**). In addition, *IL-10* and *Foxp3* gene expression were lower, while *IL-17* and *RORγT* gene expression were higher in colonic mucosa of DSS-treated mice compared with the DSS-BBR- group (*P* < 0.05, *P* < 0.01, *P* < 0.05, *P* < 0.05 respectively, **Figure 2G**). The ratio of CD4^+^IL17A^+^(Th17) cells in spleen lymphocytes were increased and the proportion of CD4^+^CD25^+^Foxp3^+^(Treg) cells in spleen lymphocytes were decreased in the DSS+BBR- group compared with DSS-BBR- group (*P* < 0.01, respectively, **Figure 2H**). BBR treatment decreased the number of CD4^+^IL17A^+^(Th17) cells and increased the number of CD4^+^CD25^+^Foxp3^+^(Treg) cells in spleen lymphocytes (*P* < 0.05, **Figure 2H**).

### BBR Changed the Structure of Gut Microbiota in UC Model Mice

**FIGURE 3:**
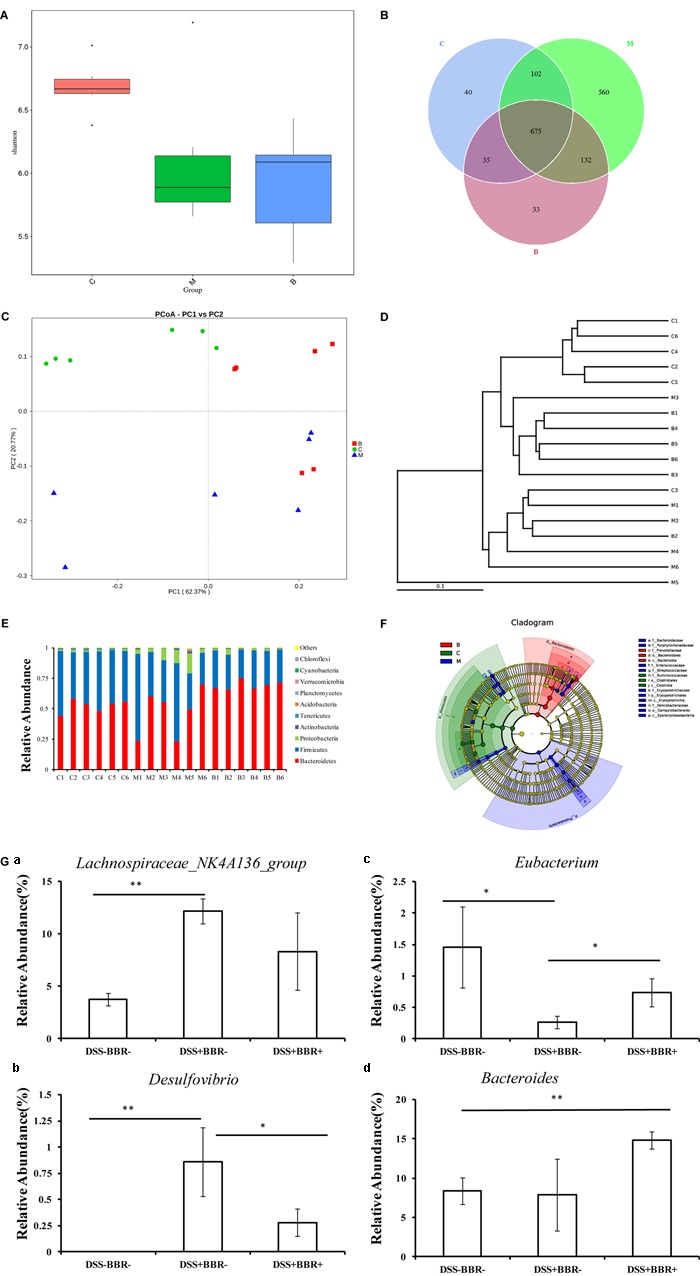
Berberine changed the structure of gut microbiota in UC model mice. **(A)** Shannon index calculated after rarefying to an equal number of sequence reads for all samples, the Shannon indexes were decreased after DSS and BBR treatment; **(B)** Venn diagram indicated the differential numbers of OTUs in each group; **(C)** PCoA score based on weighted Unifrac metrics was different in each group; **(D)** System clustering tree of gut microbiota based on weighted Unifrac metrics indicated the different beta diversity of gut microbiota in each group; **(E)** BBR treatment changed the microbial community in phylum level (bar plot); **(F)** Cladogram of gut microbiota in each group indicated the predominant microbiota in DSS+BBR+ group was *Bacteroidetes*; **(G)** The relative abundance of *Lachnospiraceae_NK4A136_group* **(a)** was increased in DSS+BBR– group compared with DSS–BBR– group whereas BBR treatment decreased the relative abundance of *Desulfovibrio* **(b)** and increased the relative abundance of *Eubacterium* **(c)** compared with DSS+BBR– group. Moreover, the relative abundance of *Bacteroides* **(d)** was increased in DSS+BBR+ group compared with DSS–BBR– group. C: DSS–BBR– group (*n* = 6). M: DSS+BBR– group (*n* = 6). B: DSS+BBR+ group (*n* = 6). Data are presented as mean ± SD. ^∗^*p* < 0.05, ^∗∗^*p* < 0.01.

A bar-coded pyrosequencing run was used to study the changes in gut microbiota from UC model mice after BBR treatment. In total, 1,454,065 useable reads and 1,577 OTUs were obtained from 18 samples. The Shannon indexes were lower in the DSS+BBR- and DSS+BBR+ groups compared with the DSS-BBR- group (**Figure 3A**). Moreover, a Venn diagram of the three groups revealed that 675 OTUs overlapped among the groups; 777 OTUs were present in the DSS-BBR- and DSS+BBR- groups; 710 in the DSS-BBR- and DSS+BBR+ groups; and 807 in the DSS+BBR- and DSS+BBR+ groups (**Figure 3B**). PCoA (principal co-ordinates analysis) showed that gut microbiota in the DSS+BBR- group were separated from the DSS-BBR- group, whereas the distance between the DSS+BBR+ and DSS-BBR- groups were closer than that between the DSS+BBR- and DSS-BBR- groups (**Figure 3C**). The system clustering tree also indicated a significant difference in each group and the distance from the DSS+BBR+ group to the DSS-BBR- group was small (**Figure 3D**).

We further investigated the gut microbiota species and their relative abundance. At the phylum level, 9 phyla could be found in all samples and the most abundant phyla in all samples were *Bacteroidetes, Firmicutes*, and *Proteobacteria* (**Figure 3E**). Conversely, *Planctomycetes* were detected in the DSS+BBR- and DSS+BBR+ groups but not in the DSS-BBR- group (**Figure 3E**). Moreover, LefSe (LDA Effect Size) analysis was used to identify biomarkers and dominant microbiota in each group. The resulting cladogram revealed that *Firmicutes* were the major microbiota in the DSS-BBR- group. *Proteobacteria, Streptococcaceae, Enterococcaceae*, and *Erysipelotrichale* were predominant intestinal flora in the DSS+BBR- group and *Bacteroidetes* were predominant in the DSS+BBR+ group (**Figure 3F**). Furthermore, the relative abundance of *Desulfovibrio* and *Lachnospiraceae_NK4A136_group* were significantly higher whereas relative abundance of *Eubacterium* was remarkably lower in the DSS+BBR- group compared with the DSS-BBR- group (**Figure 3G**). The relative abundance of *Desulfovibrio* was decreased whereas the relative abundance of *Eubacterium* was increased in the DSS+BBR+ group compared with the DSS+BBR- group (**Figure 3G**). Interestingly, the relative abundance of *Bacteroides* in the DSS+BBR+ group was significantly increased compared with the DSS-BBR- group and there were no significant differences in *Bacteroides* between the DSS+BBR- and DSS-BBR- groups (**Figure 3G**). In summary, our results showed that BBR could modulate gut microbiota composition in UC model mice.

### Modulation of Treg/Th17 Balance by BBR Following Depletion of Gut Microbiota

**FIGURE 4:**
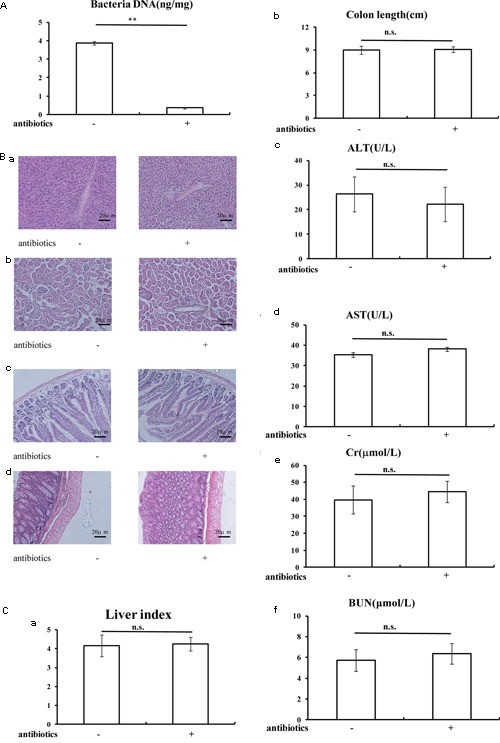
General statements of mice were not affected after antibiotics treatment. **(A)** Antibiotics treatment decreased the total DNA of gut microbiota in mice fecal sample; **(B)** H&E staining (20x) indicated the antibiotics treatment could not affected the morphology of liver **(a)**, kidney **(b)**, small intestine **(c)**, and colon **(d)**; **(C)** Liver index **(a)**, colon length **(b)**, and serum ALT **(c)**, AST **(d)**, Cr **(e)**, BUN **(f)** levels were not changed after antibiotics treatment. Data are presented as mean ± SD. ^∗^*p* < 0.05, ^∗∗^*p* < 0.01. n.s., not statistically significant.

A combination of antibiotics was used to deplete the gut microbiota followed by BBR treatment to study the effects on the Treg/Th17 balance (Elinav et al., 2011). Following the 3-week-antibiotic treatment, total DNA of gut microbiota in antibiotics-treated mice fecal samples were significantly lower than in mice that were not treated with antibiotics (*P* < 0.01, **Figure 4A**). Moreover, H&E staining showed that there were no morphological differences in liver, kidney, small intestine, or colon after antibiotics treatment compared to untreated mice (**Figure 4B**). There were no significant differences in liver index, colon length, or in serum levels of ALT, AST, Cr, and BUN after antibiotics treatment as compared to the untreated group, which indicated that antibiotics treatment depleted the gut microbiota. However, the liver and kidney function remained unaffected (**Figure 4C**).

**FIGURE 5:**
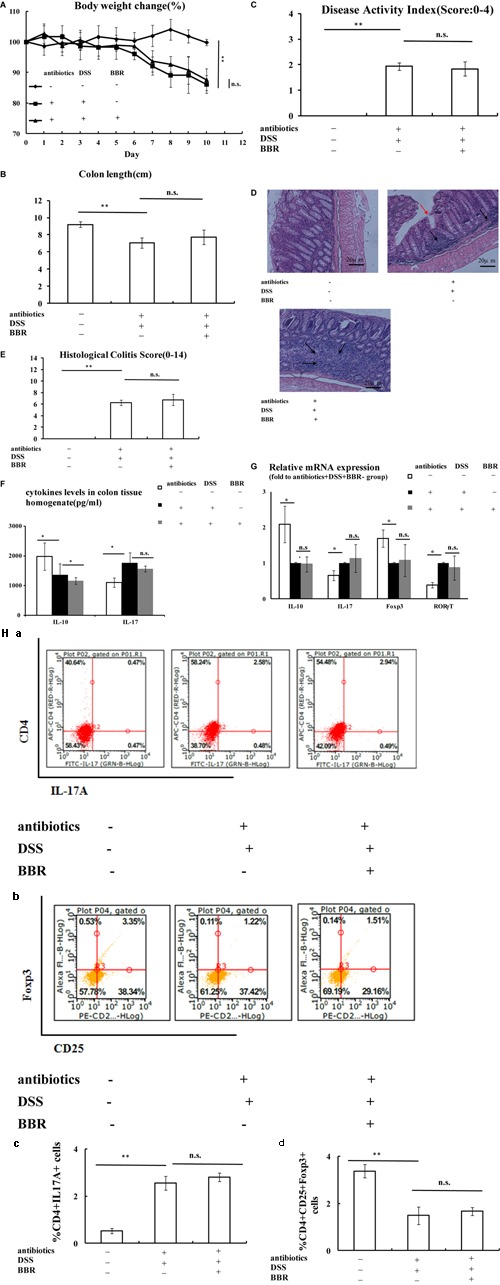
After the depletion of gut microbiota, BBR could not improve Treg/Th17 balance in DSS-induced UC model mice. **(A–C)** After the depletion of gut microbiota, BBR could not affect body weight, shorten of colon length and DAI score in DSS-induced UC model mice; **(D,E)** H&E staining (Arrows in red indicate mucosal necrosis, arrows in black indicate inflammatory cell infiltration; 20x) and histological colitis score showed that BBR could not reduce mucosal necrosis and inflammatory cell infiltration in colon after the depletion of gut microbiota; **(F)** After the depletion of gut microbiota, BBR treatment decreased *IL-10* levels in colon tissue homogenate and BBR treatment could not affect *IL-17* levels in colon tissue homogenate compared with antibiotics+DSS+BBR– group; **(G)** After the depletion of gut microbiota, BBR treatment could not affect the relative mRNA expression of *IL-10, IL-17, Foxp3* and *RORγT* in colonic mucosa compared with antibiotics+DSS+BBR– group (Fold to DSS+antibiotics+BBR– group); **(H)** After the depletion of gut microbiota, flow cytometric analysis showed that BBR treatment did not affect the numbers of CD4^+^IL17A^+^ (**a**: plot; **c**: graph) cells and CD4^+^CD25^+^Foxp3^+^ (**b**: plot; **d**: graph) in spleen compared with antibiotics+DSS+BBR– group. Antibiotics–DSS–BBR-, antibiotics+DSS+BBR–, and antibiotics+DSS+ BBR+group (*n* = 10 per group, for **A–G**). Antibiotics–DSS–BBR–, antibiotics+DSS+BBR-, and antibiotics+DSS+ BBR+group (*n* = 6 per group, **H**). Data are presented as mean ± SD. ^∗^*p* < 0.05, ^∗∗^*p* < 0.01.

In addition, following DSS treatment, antibiotics+DSS+BBR- treated mice showed significant body weight loss, shortened colon length and a high DAI score (**Figures 5A–C**, respectively) compared with mice with neither DSS nor antibiotics treatment (*P* < 0.01), whereas there were no significant differences between body weight loss, colon length, and DAI score in antibiotics+DSS+BBR+ treated mice compared with antibiotics+DSS+BBR- treated mice (**Figures 5A–C**). H&E staining also demonstrated that BBR did not relieve mucosal necrosis or infiltration of inflammatory cells triggered by DSS treatment. In addition, there was no significant difference in histological colitis score between antibiotics+DSS+BBR+ treated mice compared with antibiotics+DSS+BBR- treated mice (**Figures 5D,E**).

Consistent with the earlier observations, *IL-10* levels in colon tissue homogenate were decreased whilst *IL-17* levels in colon tissue homogenate were increased in antibiotics+DSS+BBR- treated mice compared with antibiotics-DSS-BBR- treated mice (*P* < 0.05, **Figure 5F**). Even though *IL-10* levels in colon tissue homogenate were even lower in antibiotics+DSS+BBR+ treated mice compared with antibiotics+DSS+BBR- treated mice (*P* < 0.05, **Figure 5F**), there was no significant difference between *IL-17* levels in colon tissue homogenate in antibiotics+DSS+BBR+ treated mice compared with antibiotics+DSS+BBR- treated mice (**Figure 5F**). Colonic mucosa *IL-10* and *Foxp3* expression were lower, and *IL-17* and *RORγT* expression were higher in antibiotics+DSS+BBR- treated mice compared with mice with neither antibiotics nor DSS administration (*P <* 0.05, **Figure 5G**), whereas there were no obvious differences in colonic mucosa expression of *IL-10, Foxp3, IL-17*, or *RORγT* between antibiotics+DSS+BBR- treated mice compared with antibiotics+DSS+BBR+ treated mice (**Figure 5G**). The number of CD4^+^IL17A^+^(Th17) cells in spleen lymphocytes were increased and the proportion of CD4^+^CD25^+^Foxp3^+^(Treg) cells in spleen lymphocytes were decreased in antibiotics+DSS+BBR- treated mice compared with mice in the antibiotics-DSS-BBR- group (*P* < 0.01, **Figure 5H**). There were no significant differences in the CD4^+^IL17A^+^(Th17) and CD4^+^CD25+Foxp3^+^ (Treg) cell ratio in spleen lymphocytes in antibiotics+DSS+BBR- treated mice compared with antibiotics+DSS+BBR+ treated mice (**Figure 5H**).

### BBR Fecal Transplantation Reduced UC by Improving Treg/Th17 Balance

**FIGURE 6:**
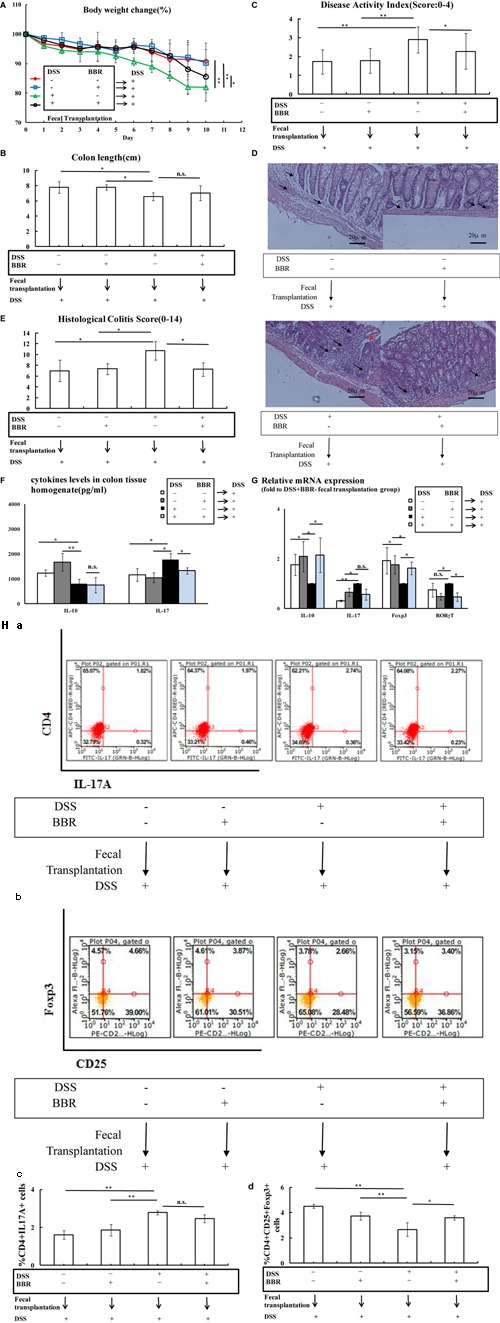
Berberine fecal transplantation reduced UC by improving Treg/Th17 balance. **(A,B)** BBR fecal transplantation reduced body weight loss and decreased loss of colon length compared with DSS+BBR– fecal transplantation group; **(C)** Mice received BBR fecal transplantation had a lower DAI score compared with DSS+BBR– fecal transplantation group; **(D,E)** H&E staining (Arrows in red indicate mucosal necrosis, arrows in black indicate inflammatory cell infiltration; 20x) and histological colitis score showed that BBR fecal transplantation reduced mucosal necrosis and inflammatory cell infiltration in colon; **(F)** BBR fecal transplantation increased *IL-10* and decreased *IL-17* levels of colon tissue homogenate compared with DSS+BBR– fecal transplantation group; **(G)** After BBR fecal transplantation, the relative mRNA expression of *IL-10* and *Foxp3* in colonic mucosa was upregulated whereas the relative mRNA expression of *IL-17* and *RORγT* in colonic mucosa was downregulated compared with DSS+BBR– fecal transplantation group (Fold to DSS+BBR– group); **(H)** Flow cytometric analysis showed that BBR fecal transplantation decreased the numbers of CD4^+^IL17A^+^(**a**: plot; **c**: graph) cells and increased the numbers of CD4^+^CD25^+^Foxp3^+^(**b**: plot; **d**: graph) in spleen compared with DSS+BBR- fecal transplantation group. DSS–BBR–, DSS-BBR+, DSS+BBR–, DSS+BBR+ fecal transplantation group (*n* = 7 per group, for **A–G**). DSS–BBR–, DSS-BBR+, DSS+BBR–, DSS+BBR+ fecal transplantation group (*n* = 6 per group, **H**). Data are presented as mean ± SD. ^∗^*p* < 0.05, ^∗∗^*p* < 0.01.

Accumulated clinical studies have shown that fecal microbiota transplantation (FMT) could treat UC by modifying the gut microbiota (Borody et al., 2003; Anderson et al., 2012; Kunde et al., 2013; Moayyedi et al., 2015). To further confirm that BBR could improve the Treg/Th17 balance in the UC mice model by regulating the gut microbiota, gut microbiota of DSS-BBR-, DSS-BBR+, DSS+BBR-, and DSS+BBR+ treated mice were transferred into DSS-induced UC mice. UC mice that received fecal transplants from DSS-BBR-, DSS-BBR+ and DSS+BBR+ treated mice showed reduced body weight loss and DAI score compared with UC mice that received fecal matter from DSS+BBR- mice (**Figures 6A,C**). In addition, UC mice receiving DSS-BBR- and DSS-BBR+ fecal transplantations had a significantly increased colon length as compared with UC mice receiving DSS+BBR- treated mice fecal transplantation (**Figure 6B**). H&E staining showed that mucosal necrosis and infiltration of inflammatory cells were reduced in UC mice that received fecal matter from DSS-BBR-, DSS-BBR+, and DSS+BBR+ treated mice as compared with UC mice that received fecal matter from DSS+BBR- treated mice. This was evidenced by lower histological colitis scores compared with UC mice that received fecal matter from DSS+BBR- treated mice (*P* < 0.05, **Figures 6D,E**). In addition, *IL-10* levels in colonic tissue homogenate were increased in UC mice that received fecal transplants from DSS-BBR- and DSS-BBR+ treated mice compared to those that received fecal transplants from DSS+BBR- treated mice (*P* < 0.05, *P* < 0.01, respectively, **Figure 6F**). However, *IL-17* levels in colonic tissue homogenate was decreased in UC mice that received DSS-BBR-, DSS-BBR+, and DSS+BBR+ treated mice fecal transplantation as compared to UC mice that received DSS+BBR- treated mice fecal transplantation (*P* < 0.05, respectively, **Figure 6F**). *IL-10* and *Foxp3* gene expression in colonic mucosa were up-regulated in UC mice that received fecal transplants from DSS-BBR- DSS-BBR+, and DSS+BBR+ treated mice as compared to those that received fecal matter from DSS+BBR- treated mice (*P* < 0.05, respectively, **Figure 6G**). *IL-17* expression was down-regulated in UC mice given fecal matter from DSS-BBR- and DSS-BBR+ treated mice compared to UC mice administered feces from DSS+BBR- treated mice (*P* < 0.01, *P* < 0.05, respectively, **Figure 6G**). Moreover, *RORγT* gene expression in colonic mucosa was down-regulated in UC mice given feces from DSS-BBR+ and DSS+BBR+ treated mice compared to those given feces from DSS+BBR- treated mice (*P* < 0.05, **Figure 6G**). The number of CD4^+^IL17A^+^(Th17) cells in spleen lymphocytes were decreased in UC mice receiving fecal transplantation from DSS-BBR- and DSS-BBR+ treated mice as compared to UC mice receiving fecal transplantation from DSS+BBR- treated mice (*P* < 0.01, **Figure 6H**), whereas the proportion of CD4^+^CD25^+^Foxp3^+^(Treg) cells of spleen were decreased in UC mice receiving fecal transplantation from DSS-BBR-, DSS-BBR+, and DSS+BBR+ treated mice as compared to UC mice receiving fecal transplantation from DSS+BBR- treated mice (*P* < 0.01, *P* < 0.01, *P* < 0.05, respectively, **Figure 6H**).

## Discussion

In the present study, the effect of BBR on DSS-induced UC was studied *in vivo.* Our results indicated a significant effect of BBR on UC, evidenced by a reduction in body weight loss, DAI score, mucosal necrosis, and infiltration of inflammatory cells as well as shortening of colon length. This work is in line with an earlier study (Shu et al., 2006).

Previous study has demonstrated that BBR could inhibit Th17 response in T-cell-mediated autoimmune diseases (Yang et al., 2013). It has also been reported that BBR could inhibit Th1/Th17 differentiation in UC model mice (Li et al., 2015). We further investigated the effect of BBR on Treg/Th17 balance, one of the important mechanisms in UC progression. Th17 cells differentiate from naïve CD4^+^T cells through stimulation by transforming growth factor *(TGF)*-β and *IL-6*. This process is mediated under the transcriptional regulation of retinoic acid-related orphan receptor (*RORγT*) (Ivanov et al., 2007; Ziegler and Buckner, 2009). Th17 cells produce pro-inflammatory cytokines including *IL-17, IL-21, IL-22, IL-23*, and *IL-25*, which contribute to the progression of UC (Korn et al., 2009; Weaver et al., 2013). Tregs could be distinguished into natural Tregs (nTregs) and inducible Tregs (iTregs) due to their different origins. nTregs are derived from the thymus with the function of suppressing the innate immune response (van Wijk and Cheroutre, 2010), whereas iTregs differentiate from naïve CD4^+^T cells in the periphery when stimulated by specific antigens and they are crucial in the suppressive control of adaptive immunity (van Wijk and Cheroutre, 2010). The differentiation of both iTregs and nTregs are regulated by the transcription factor forkhead box P3 (*Foxp3*). Mature Tregs express high levels of *Foxp3* (Fontenot et al., 2003; Bacchetta et al., 2006). Moreover, both iTregs and nTregs could secrete inhibitory cytokines including *IL-10, IL-35*, and *TGF-β*, which could suppress both adaptive and innate immune responses (Jonuleit and Schmitt, 2003). Studies have shown that increased Th17 cells produced high levels of *IL-17*, promoting inflammation. However, differentiation of Tregs was inhibited, triggering the reduction of immunosuppressive cytokines including *TGF-β* and *IL-10* in the mucosa (Fujino et al., 2003; Sugihara et al., 2010; Schiering et al., 2014). Furthermore, improving Treg/Th17 balance contributed to the re-establishment of intestinal immune homeostasis and reduced UC (Bai et al., 2009; Lee et al., 2015). In this study, BBR treatment improved the Treg/Th17 balance in DSS-induced UC model, consistent with previous studies.

It has been demonstrated that *Bacteroides* could interact with Treg to promote *IL-10* production by producing metabolites (Smith et al., 2013; Luo et al., 2017). Colonization of segmented filamentous bacteria could induce Th17 cell differentiation in the intestinal lamina propria (Ivanov et al., 2009). We also investigated changes in microbiological composition using high-throughput sequencing. Our results showed that DSS treatment could reduce the alpha diversity of the gut microbiota community based on a lower Shannon index compared with DSS-BBR- treated mice. Moreover, PCoA analysis and the system clustering tree showed significant distances between each group, consistent with the previous study that DSS treatment could change the beta diversity of the gut microbiota community (Yang et al., 2017). Interestingly, the distances in PCoA analysis and the system clustering tree were close between the DSS+BBR+ and DSS-BBR- groups. Our results also showed that BBR could decrease the relative abundance of *Desulfovibrio* and increase *Eubacterium* strains after DSS treatment. In agreement with a previous study, *Desulfovibrio* bacterial species were increased in UC and *Desulfovibrio* were harmful to colonic epithelial cells (Rowan et al., 2010). *Eubacterium limosum* could ameliorate experimental colonic inflammation by producing butyrate to increase mucosal integrity (Kanauchi et al., 2006). Additionally, our results also revealed a significant increase of *Bacteroides* after BBR treatment.

We also investigated whether gut microbiota are involved in the regulation of the Treg/Th17 balance by BBR in UC. The depletion of gut microbiota through antibiotics treatment is commonly used in order to understand the role of gut microbiota (Elinav et al., 2011; Hoban et al., 2017). In our study, analysis of fecal total DNA showed that gut microbiota were depleted after treatment with a combination of antibiotics. Due to the hepatotoxicity and nephrotoxicity of antibiotics, we also measured the long-term effect of antibiotics on liver and kidney function in mice. The use of antibiotics did not affect serum ALT, AST, Cr, or BUN levels, nor did it affect the histological profiles of the liver, kidney, small intestine, or colon. It was also observed that BBR had a decreased effect on UC following depletion of gut microbiota, indicating that BBR treatment does not influence Treg/Th17 balance after depletion of gut microbiota.

Fecal transplantation is a novel method for studying the relationship between herbs and microbiota. Research has shown that mice administered *Ganoderma lucidum* fecal transplants had reduced high fat diet-induced obesity, indicating that gut microbiota is a key factor for *G. lucidum* in reducing obesity (Chang et al., 2017). In our study, fecal transplantation was carried out in order to assess the role played by gut microbiota during treatment of UC with BBR. Our findings indicated that fecal samples from BBR treated mice resulted in reduced UC and modulated Treg/Th17 balance.

In the present study, we first determined Treg and Th17 activities in UC model mice treated with BBR. Our results indicated that BBR influenced the Treg/Th17 balance in UC model mice. Secondly, we found that the effect of BBR on the Treg/Th17 balance in UC model mice was decreased after gut microbiota were depleted through antibiotics treatment. Finally, when fecal samples from mice that received BBR treatment were transplanted into UC model mice, we found that the imbalance of Treg/Th17 in recipient mice was improved. In conclusion, gut microbiota plays an important role during UC treatment as it affects the efficacy of BBR. The mechanism for this effect could be related to regulation of the Treg/Th17 balance.

## Author Contributions

HC wrote the manuscript. YC, XC, JiL, LW, HC, and SZ conducted animal experiments. BJ, JuL, YC, and XZ finished molecular bioassays. YB and PZ provided technical guidance for the whole work. All the authors approved the final draft.

## Conflict of Interest Statement

The authors declare that the research was conducted in the absence of any commercial or financial relationships that could be construed as a potential conflict of interest.
